# Supplementary material for: Chromothripsis during telomere crisis is independent of NHEJ, and consistent with a replicative origin
Source: Genome Res. 2019 May;29(5):737–49. doi: 10.1101/gr.240705.118 (PMC6499312; doi:10.1101/gr.240705.118)
Supplement: Supplemental Material [file supp_gr.240705.118_Supplemental_file_1.zip › contigs/annotated_contigs/DB114/contig.2.DB114_length_1098_mean_cov_10.7559198543.docx]

**DB114_length_1098_mean_cov_10.7559198543**

```
GTTTGTAAAAATTACCTATTTTTGTTCCCCACCGTGAAATTTTTTATTGTGGAAGTAACCTGTGCTCGTGGAGTTTTTATGGTTCAGAA
 >chr21:46380412-46381026 + E=0e+00
GTGCCTGCAGTGGTATGTGAAATCCTTCCAGGCCTTGCCCGCGGTGAAAACAGTGTCTGCGCATAGCGGCGTGGGTGTGGGCAGGGCCC

CTGGTGTCCCCAGACCCTTTCAGGCCTGTGGCTCCCTCTTTTCTGAGGTCAGTCTTGCCACGTGCTCCCAGCTGCACGTCTCCGCAGGC

TGAAAGCCACACTTCCCACTCAGGTTGTTTTGGAGTATCTCCTTATTTTTCGTGATGCTGGATATTTGCCAGTGTTTTTCTAAGATTGT

TTCTCGTCAAAATTATTTTGTATTTTTCGGTTATCTTTTTAGTGTGATTCTGCTGACTTTGTGAAATTAATTTGGAATATTTTCTTTTT

TATGTTATCTGCTTTATTTTTTCCTTGGAGGTTTTAAAGAAAATGTATGTTTTTCAGAGGTAGGACCTAGAAAACTTTGTCAGTTTCTT

TTGTGATTATTAGTTTTATGGTTTCTATCCCTTTTTGGATATTTCTTAGGGAAATGATTCATTCCTCCATTTTC|TTTTTC|CCCAAAC
                                                                           >chr21:46376
GGCCTGTCCCACGATGCATTTCAGCTTGCATTCACCCAGAAGGCGTGGCGAGAAGAACCTCGGCACCACGAGATGCTGCTGGCATGTGC
312-46376802 + E=1e-279
CGCTTCAGCTGGTGACCTGGGTGGTGTTGGCAGCATCTGAGTGGCTCTGTAGGCCGCAGCATCTCCCGGCACCCGAGCCCGAGATGAAG

CAGCACGTGCCAGCAGCATCGCGTAGTGTGTTCTGTCCTGAGCCTCATGCAGATCTTTGTGGAGTGAGAATCAAGGCTTGTTGATGGCG

TGCACGGAAAAGCCGGTCTCATGCTGATTTTCCTTAGGGTCCATCAGAACACGTGCCCCGTGAACCGTGTCACCCCACAGCCCAACTCT

GGTGAGCCTGGTTCTGTGTCTGCTGACCTGGTGAGGGCTGGGGACCAGGTGAGTTGCCTGCGGCACAGGCGTGCTGTGTAGCTGCCATG

TGTGGCTTCCTCCACTGAGGCTGGTTTACCGC
```
